# Supplementary material for: Multi-site fungicides suppress banana Panama disease, caused by Fusarium oxysporum f. sp. cubense Tropical Race 4
Source: PLoS Pathog. 2022 Oct 20;18(10):e1010860. doi: 10.1371/journal.ppat.1010860 (PMC9584521; doi:10.1371/journal.ppat.1010860)
Supplement: S1 Fig — (PDF) [file ppat.1010860.s001.pdf]

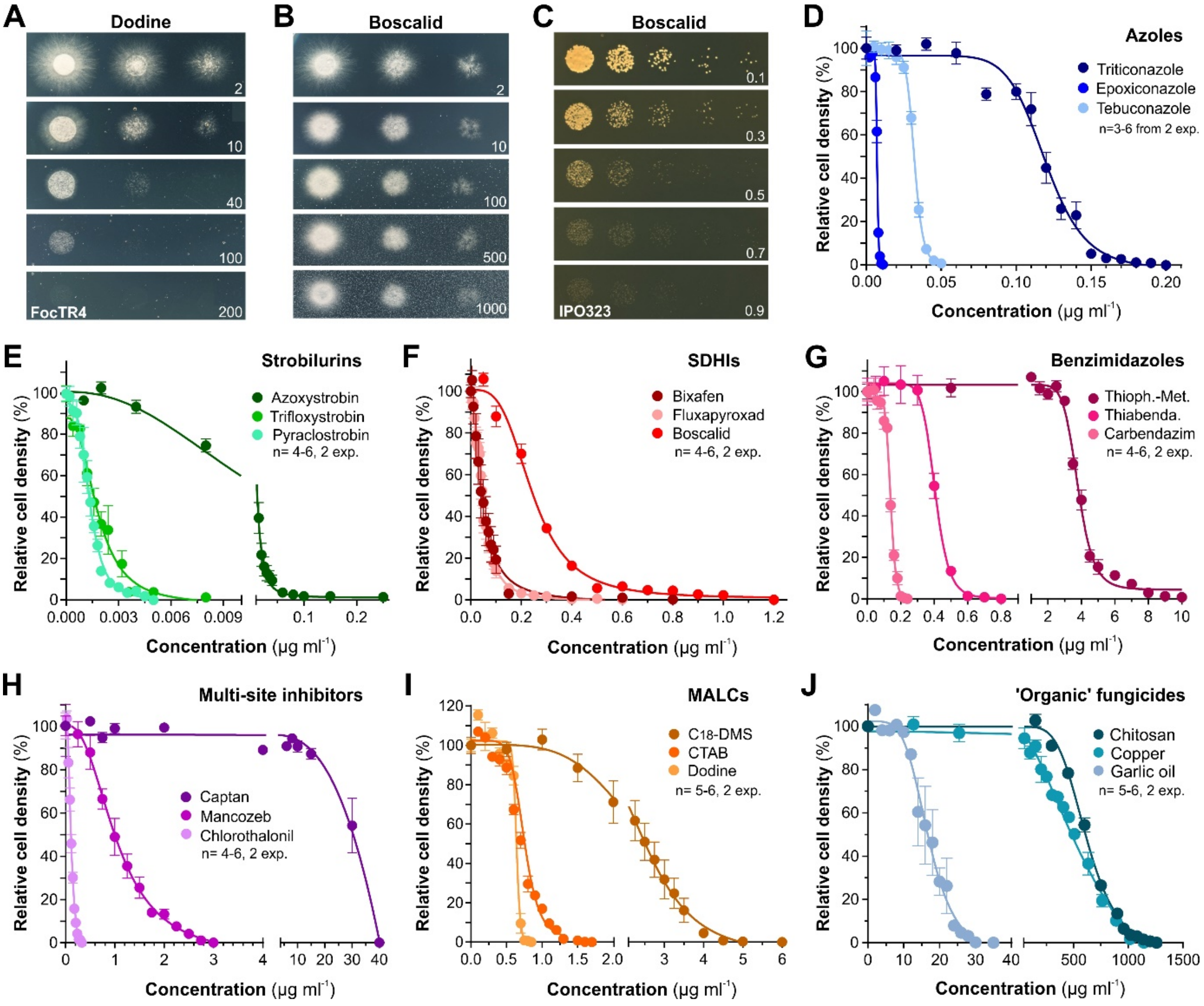

S1_Fig. The effect of fungicides on growth of *Z. tritici* strain IPO323 on solid medium.

A FocTR4 colonies on PDA plates, supplemented with various concentrations of dodine, after 2 days growth at 25°C. Note that the corona of hyphae disappears at lower concentrations as compared with the central cells in the colony centre. Fungicide concentrations in µg ml^{-1} given in lower right corner.

B FocTR4 colonies on PDA plates, supplemented with various concentrations of boscalid, after 2 days growth at 25°C. Fungicide concentrations in µg ml^{-1} given in lower right corner.

C Examples of *Z. tritici* colony formation after 5 days growth at 18°C on YPD agar plates, supplemented with the succinate dehydrogenase inhibitor boscalid. Fungicide concentrations, given as µg ml^{-1}, shown in lower right corner.

D - J Growth curves of *Z. tritici* on YPD agar plates, supplemented with various fungicides, measured after 5 days at 18°C. Each fungicide class is represented by 3 compounds. SDHIs, succinate dehydrogenase inhibitors; MALCs, mono-alkyl

lipophilic cations. See also Table S2 for estimated concentrations at 50% (EC_{50}); 90% (EC_{90})) and >99.5% (MIC) growth inhibition.

All data points in (**D**-**J**) are given as mean ± SEM of 6 measurements from 2 independent experiments. Non-linear regression in (**D**-J) used GraphPad Prism 6, equations "[Inhibitor] vs. response - variable slope (four parameters)".
