# Supplementary material for: Multi-site fungicides suppress banana Panama disease, caused by Fusarium oxysporum f. sp. cubense Tropical Race 4
Source: PLoS Pathog. 2022 Oct 20;18(10):e1010860. doi: 10.1371/journal.ppat.1010860 (PMC9584521; doi:10.1371/journal.ppat.1010860)
Supplement: S2 Fig — (PDF) [file ppat.1010860.s002.pdf]

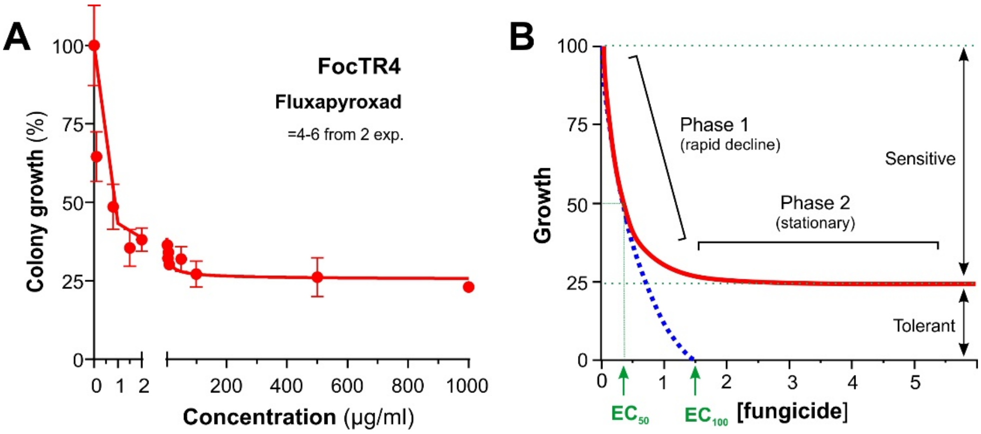

S2_Fig. Two phase response of FocTR4 colony formation to fluxapyroxad.

A Colony growth on fluxapyroxad-containing agar plates (data were taken from Fig. 3).

B Diagram illustrating the 2 phases of response to the fungicide. 75% of the population is susceptible to the fungicide and probably dies at a low MIC (blue dotted line). However, this is not visible in the dose-response curve, as the remaining 25% are tolerant and survive the fungicide treatment (red curve). The combined behaviour of both populations results in a "two-phased" fungicide response curve (phases indicated in graph).
