# Supplementary material for: Multi-site fungicides suppress banana Panama disease, caused by Fusarium oxysporum f. sp. cubense Tropical Race 4
Source: PLoS Pathog. 2022 Oct 20;18(10):e1010860. doi: 10.1371/journal.ppat.1010860 (PMC9584521; doi:10.1371/journal.ppat.1010860)
Supplement: S3 Fig — (PDF) [file ppat.1010860.s003.pdf]

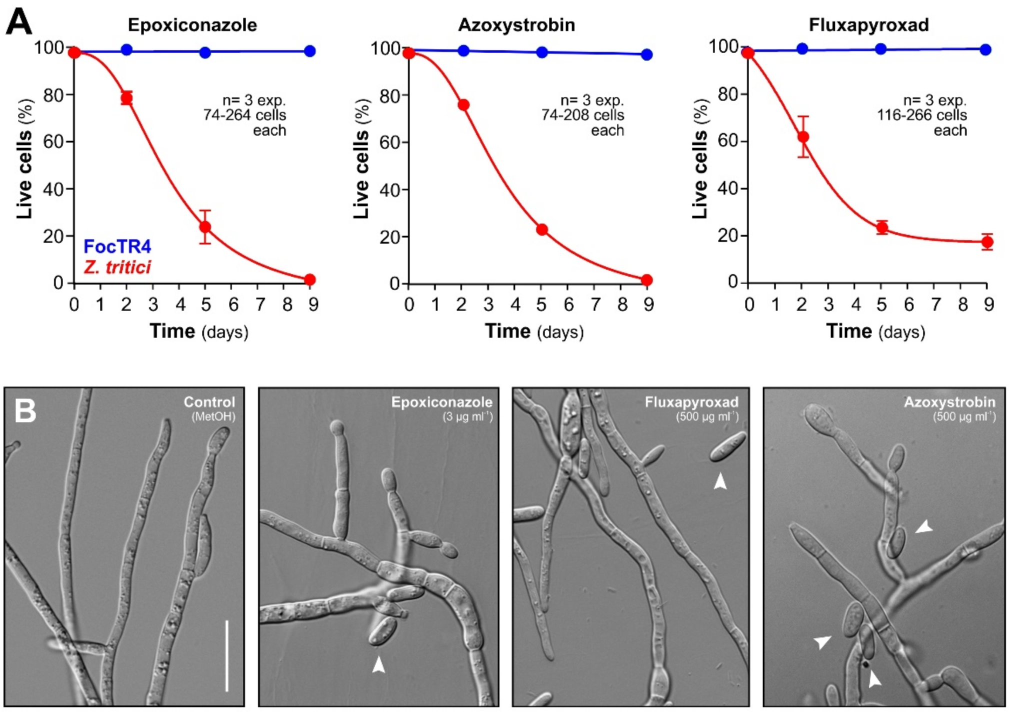

S3_Fig. Mortality of FocTR4 and *Z. tritici* IPO323 in liquid cultures (PDB) and appearance of persister hyphae.

A Relative number of living cells, identified by LIVE/DEAD staining, in FocTR4 and Z*. tritici* IPO323 grown in liquid cultures, supplemented with epoxiconazole (3 µg ml^{-1}), fluxapyroxad and azoxystrobin (both 500 µg ml^{-1}).

B Morphology of fungicide persister cells, grown in liquid medium cultures. FocTR4 microconidia were incubated for 2 days in PDB, supplemented with epoxiconazole (3 µg ml^{-1}), fluxapyroxad (500 µg ml^{-1}), azoxystrobin (500 µg ml^{-1}) or equivalent amounts of the control solvent methanol (Control). Scale bar= 20 µm
