# Supplementary material for: Multi-site fungicides suppress banana Panama disease, caused by Fusarium oxysporum f. sp. cubense Tropical Race 4
Source: PLoS Pathog. 2022 Oct 20;18(10):e1010860. doi: 10.1371/journal.ppat.1010860 (PMC9584521; doi:10.1371/journal.ppat.1010860)
Supplement: S4 Fig — (PDF) [file ppat.1010860.s004.pdf]

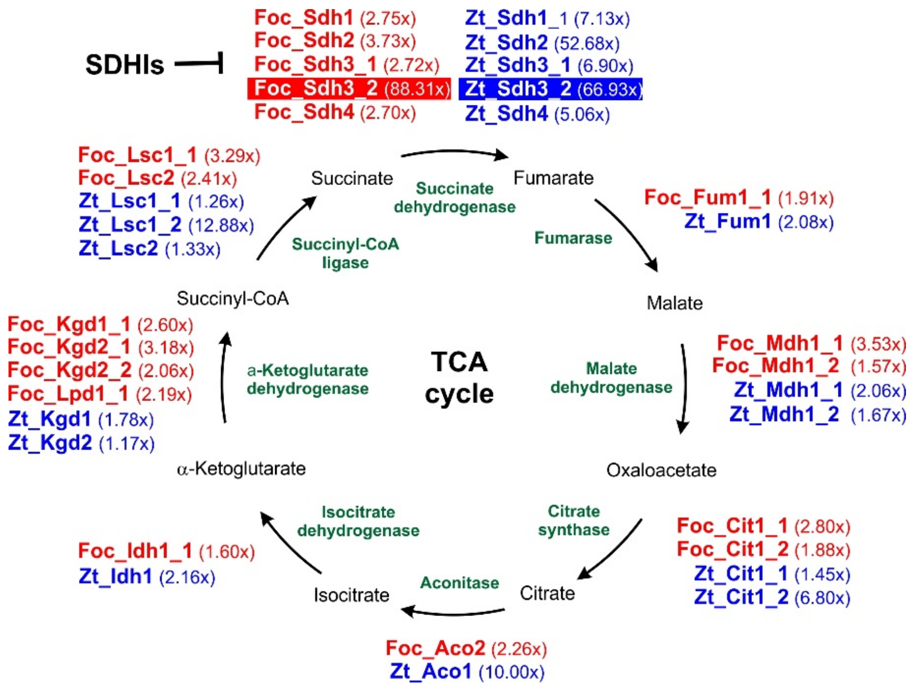

S4_Fig. Expression of TCA cycle genes in fluxapyroxad-treated FocTR4 and IPO323 cells. FocTR4 genes are shown in red, IPO323 genes are in blue. Mean up-regulation is given in parenthesis. Note that Sdh3-subunits are up-regulated in both fungi (indicated). Also note that enzymes involved in the conversion of α-ketoglutarate to succinyl-CoA are 3-times more strongly expressed in FocTR4. For accession numbers of all genes see S4_Table, S5_Table and S6_Table.
