# Supplementary material for: Multi-site fungicides suppress banana Panama disease, caused by Fusarium oxysporum f. sp. cubense Tropical Race 4
Source: PLoS Pathog. 2022 Oct 20;18(10):e1010860. doi: 10.1371/journal.ppat.1010860 (PMC9584521; doi:10.1371/journal.ppat.1010860)
Supplement: S5 Fig — (PDF) [file ppat.1010860.s005.pdf]

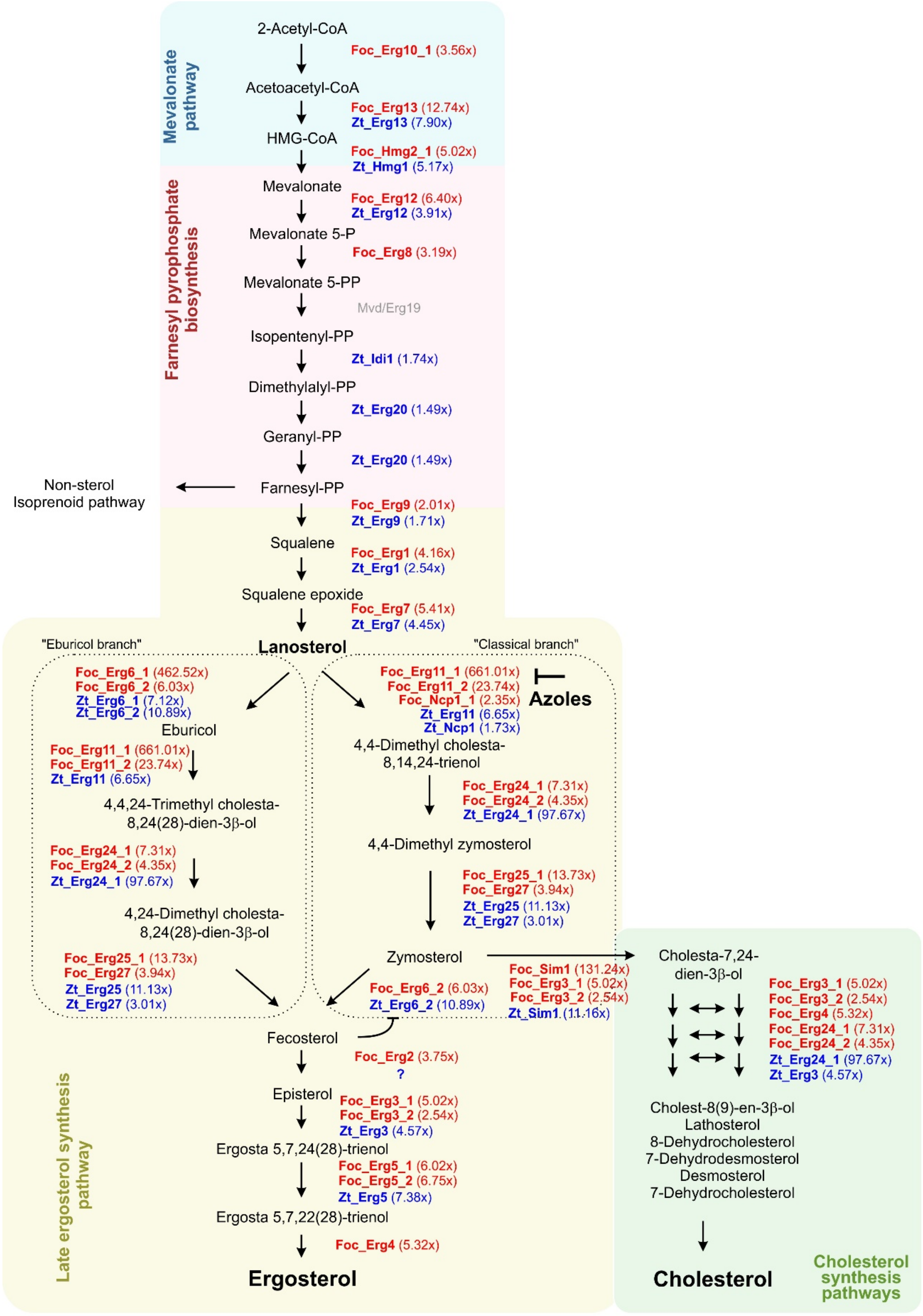

?: Unclear as Zt_Erg2 does not exist in data base

S5_Fig. Expression of genes of the ergosterol biosynthesis pathway in epoxiconazole-treated FocTR4 and IPO323 cells.

Induction of genes in the ergosterol biosynthesis pathway by epoxiconazole in FocTR4 (red) and *Z. tritici* IPO323 (blue) is indicated in parenthesis. Note that late ergosterol synthesis pathway genes are strongly up-regulated in FocTR4. Note also that FocTR4 cells strongly express a putative 3-β-hydroxysteroid-Δ(8),Δ(7)-isomerase (Sim1), absent from *S. cerevisiae*, but has significant homology to orthologues in human cells (BLAST P= 5e-47) which participates in cholesterol synthesis (Braverman *et al.*, 1999). For accession numbers of all genes see S4_Table and S5_Table.
