# Supplementary material for: Multi-site fungicides suppress banana Panama disease, caused by Fusarium oxysporum f. sp. cubense Tropical Race 4
Source: PLoS Pathog. 2022 Oct 20;18(10):e1010860. doi: 10.1371/journal.ppat.1010860 (PMC9584521; doi:10.1371/journal.ppat.1010860)
Supplement: S6 Fig — (PDF) [file ppat.1010860.s006.pdf]

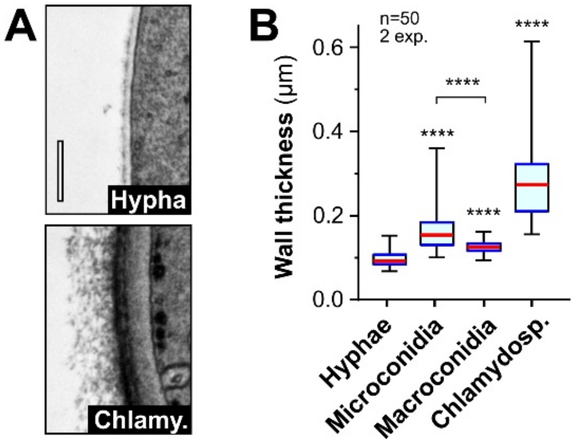

S6_Fig. Cell wall thickness in FocTR4 morphotypes.

A Cell wall of chlamydospore (lower panel) and a hypha (upper panel). Scale bar= 0.5 µm.

B Thickness of the inner cell wall in all FocTR4 morphotypes.

Data in (**B**) are non-normally distributed (Shapiro-Wilk test, P<0.05) and are shown as Whiskers' plots with 25/75 percentiles (blue line) and median (red line); statistical comparison used Mann-Whitney testing; **** = significant difference to hyphae or another data set (bracket) at two-tailed P<0.0001; sample sizes are indicated in graphs.
