# Supplementary material for: Multi-site fungicides suppress banana Panama disease, caused by Fusarium oxysporum f. sp. cubense Tropical Race 4
Source: PLoS Pathog. 2022 Oct 20;18(10):e1010860. doi: 10.1371/journal.ppat.1010860 (PMC9584521; doi:10.1371/journal.ppat.1010860)
Supplement: S7 Fig — (PDF) [file ppat.1010860.s007.pdf]

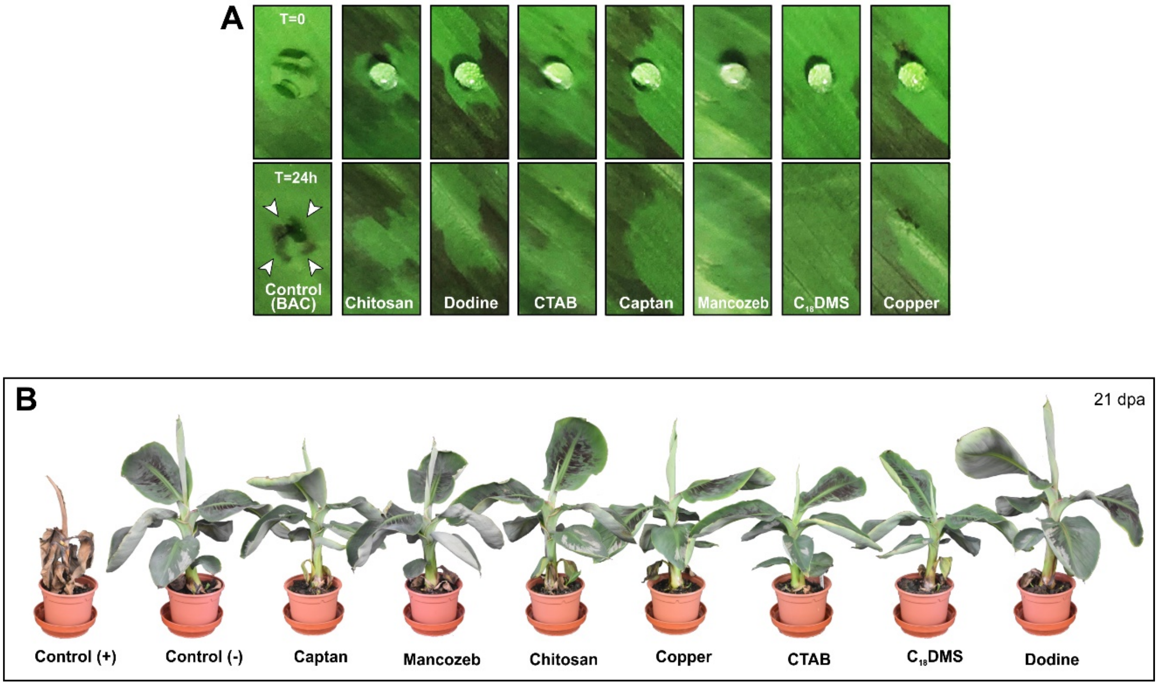

S7_Fig. Phytotoxicity of fungicide solutions in bananas.

A Test for fungicide phytotoxicity on attached banana leaves. BAC was used as a positive control (Control(+); necrosis indicated by arrowheads). Upper row shows droplets at moment of application (T=0h), lower row shows same tissue after 24h incubation at 27^{0}C.

B Test for phytotoxicity of fungicides in entire plants. Fungicide containing solutions (50 ml) were applied to the root system twice (at 0 days and 7 days). Plants are shown for 21 days at 27ºC after application (dpa) of the first fungicide treatment. Note that this temperature reflects optimal growth conditions in the field (Varma & Bebber, 2019).

Concentrations used: 50 mg ml^{-1} BAC (Control in **a**, Control (+) in **b**), 20 µg ml^{-1} captan, and 200 µg ml^{-1} for dodine, CTAB, mancozeb, C_{18}DMS and LMW chitosan (applied as 333 µg ml^{-1} lactate salt), and copper (applied as 786 µg ml^{-1} copper(II) sulfate pentahydrate).
