# Supplementary material for: Multi-site fungicides suppress banana Panama disease, caused by Fusarium oxysporum f. sp. cubense Tropical Race 4
Source: PLoS Pathog. 2022 Oct 20;18(10):e1010860. doi: 10.1371/journal.ppat.1010860 (PMC9584521; doi:10.1371/journal.ppat.1010860)
Supplement: S8 Fig — (PDF) [file ppat.1010860.s008.pdf]

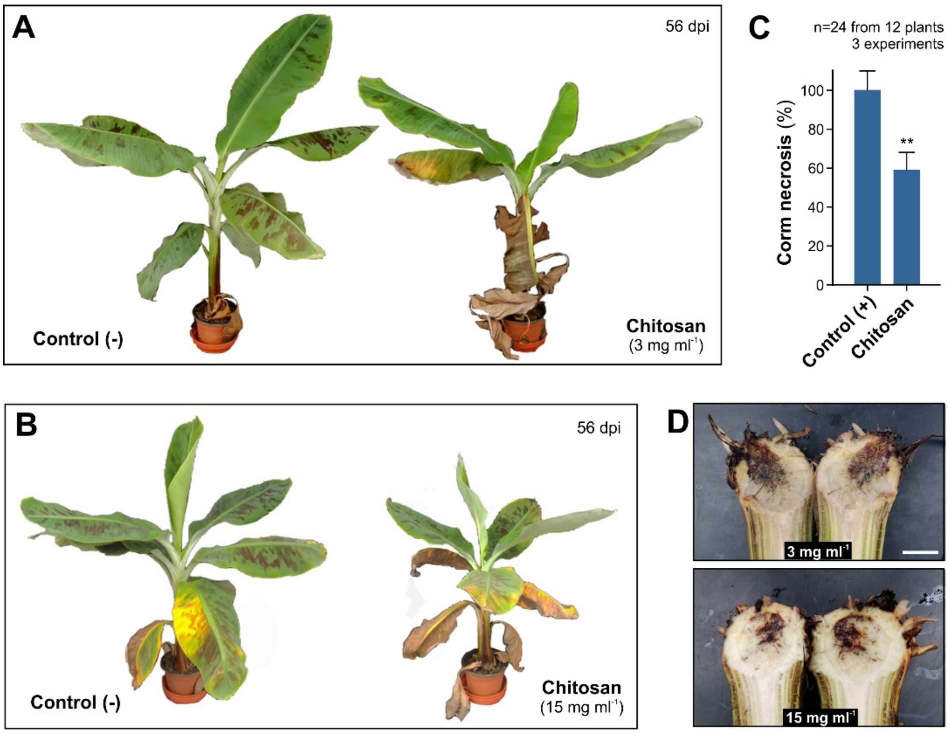

S8_Fig. Panama disease symptoms in infected chitosan-treated plants.

A Whole-plant symptoms of Panama disease at 56 days after root inoculation with chlamydospores (as described), followed by 2 treatments (day 0 and day 7) with 3000 µg ml^{-1} LMW chitosan or water (control)

B Whole-plant symptoms of Panama disease at 56 days after root inoculation with chlamydospores (as described), followed by 2 treatments (day 0 and day 7) with 15000 µg ml^{-1} LMW chitosan or water (Control).

C Quantitative assessment of corm tissue necrosis following inoculation with FocTR4 chlamydospores and 2 treatments (day 0 and day 7) with 3000 µg ml^{-1} LMW chitosan (as in a.). Banana corm necrosis was analysed 56 days after the first treatment (treatment 2 at day 7). Statistical testing used Student's t-test, **= error probability of P= 0.0038.

D Corm necrosis in bananas at 56 days after root inoculation with chlamydospores, followed by 2 treatments with 3000 µg ml^{-1} LMW chitosan and 15000 µg ml^{-1} LMW. Scale bar= 2 cm.
