# Supplementary material for: Multi-site fungicides suppress banana Panama disease, caused by Fusarium oxysporum f. sp. cubense Tropical Race 4
Source: PLoS Pathog. 2022 Oct 20;18(10):e1010860. doi: 10.1371/journal.ppat.1010860 (PMC9584521; doi:10.1371/journal.ppat.1010860)
Supplement: S1 Table — (PDF) [file ppat.1010860.s009.pdf]

S1_Table Bioinformatic tools used in this study

Programme	Web address	Reference
**FastP v0.20.1**	https://github.com/OpenGene/fastp	[1]
**FastQC 0.11.4**	www.bioinformatics.babraham.ac.uk/projects/fastqc	none
**FastQScreen 0.5.2**	bioinformatics.babraham.ac.uk/projects/fastq_screen	[2]
**BWA-mem 0.7.15**	github.com/lh3/bwa	[3]
**Picard 2.12.1**	broadinstitute.github.io/picard/	none
**GATK 3.8**	https://gatk.broadinstitute.org/hc/en-us	[4]
**IGV 2.4.10**	software.broadinstitute.org/software/igv	[5]
**Cutadapt 1.13**	cutadapt.readthedocs.io/en/stable/guide.html	[6]
**TopHat2 2.1.1**	ccb.jhu.edu/software/tophat/index.shtml	[7]
**HTSeq-count 0.10.0**	htseq.readthedocs.io/en/release_0.11.1/count.html	[8]
**DESeq2 1.14.1**	bioconductor.org/packages/devel/bioc/vignettes/DESeq2/ inst/doc/DESeq2.html	[9]
**BlastP 2.12**	https://blast.ncbi.nlm.nih.gov/Blast.cgi?PAGE=Proteins	[10]
**Interproscan 5**	https://www.ebi.ac.uk/interpro/	[11]
**TCDB**	http://www.tcdb.org/	[12]
**SignalP 5.0**	cbs.dtu.dk/services/SignalP	[13]
**TargetP 2.0**	https://services.healthtech.dtu.dk/service.php?TargetP-2.0	[14]
**DeepMito**	http://busca.biocomp.unibo.it/deepmito/	[15]
**Quest Calculate**	https://www.aatbio.com/tools/calculate-peptide-and-protein-molecular-weight-mw	none
**SIFT**	https://sift.bii.a-star.edu.sg/	[16]
**EMBOSS Needle 6.6.0**	ebi.ac.uk/Tools/psa/emboss_needle	[17]
**ClustalOmega 1.2.4**	ebi.ac.uk/Tools/msa/clustalo	[18]
**MEGA5.2**	https://mega.software.informer.com/5.0/	[19]

References:

1. Chen S, Zhou Y, Chen Y, Gu J. fastp: an ultra-fast all-in-one FASTQ preprocessor. Bioinformatics. 2018;34: i884-i890. https://doi.org/10.1093/bioinformatics/bty560. PMID: 30423086

2. Wingett S, Andrews S. 2018. FastQ Screen: A tool for multi-genome mapping and quality control. F1000Res. 2018;7: 1338. https://doi.org/10.12688/f1000research.15931.2. PMID: 30254741

3. Li H, Durbin R. Fast and accurate short read alignment with Burrows-Wheeler transform. Bioinformatics. 2009;25: 1754-1760. https://doi.org/10.1093/bioinformatics/btp324. PMID: 19451168

4. Van der Auwera G, Carneiro M, Hartl C, Poplin R, Del Angel G, Levy-Moonshine A, et al. From FastQ data to high confidence variant calls: the Genome Analysis Toolkit best practices pipeline. Curr Protoc Bioinformatics. 2013;43: 11.10.1-11.10.33. https://doi.org/10.1002/0471250953.bi1110s43. PMID: 25431634

5. Robinson J, Thorvaldsdottir H, Winckler W, Guttman M, Lander E, Getz G et al.. Integrative genomics viewer. Nat Biotechnol 2011;29: 24-26. https://doi.org/10.1038/nbt.1754. PMID: 21221095

6. Martin M. Cutadapt removes adapter sequences from high-throughput sequencing reads. EMBnet J. 2011;17: 10-12. https://doi.org/10.14806/ej.17.1.200.

7. Kim D, Pertea G, Trapnell C, Pimentel H, Kelley R, Salzberg S. TopHat2: accurate alignment of transcriptomes in the presence of insertions, deletions and gene fusions. Genome Biol. 2013; 14: R36. https://doi.org/10.1186/gb-2013-14-4-r36. PMID: 23618408

8. Anders S, Pyl P, Huber W. HTSeq--a Python framework to work with high-throughput sequencing data. Bioinformatics. 2015;31: 166-169. https://doi.org/10.1093/bioinformatics/btu638. PMID: 25260700

9. Love M, Huber W, Anders S. Moderated estimation of fold change and dispersion for RNA-seq data with DESeq2. Genome Biol. 2014;15: 550. https://doi.org/10.1186/s13059-014-0550-8. PMID: 25516281

10. Altschul S, Gish W, Miller W, Myers E, Lipman D. Basic local alignment search tool. J Mol Biol. 1990;215: 403-410. https://doi.org/10.1016/S0022-2836(05)80360-2. PMID: 2231712

11. Jones P, Binns D, Chang H, Fraser M, Li W, McAnulla C et al. InterProScan 5: genome-scale protein function classification. Bioinformatics. 2014;30: 1236-1240. https://doi.org/10.1093/bioinformatics/btu031. PMID: 24451626

12. Saier M, Reddy V, Moreno-Hagelsieb G, Hendargo K, Zhang Y, Iddamsetty V, et al. The Transporter Classification Database (TCDB): 2021 update. Nucleic Acids Res. 2021;49: D461-D467. https://doi.org/10.1093/nar/gkaa1004. PMID: 33170213

13. Almagro Armenteros J, Tsirigos K, Sonderby C, Petersen T, Winther O, Brunak S, et al. SignalP 5.0 improves signal peptide predictions using deep neural networks. Nat. Biotechnol. 2019a;37: 420-423. https://doi.org/10.1038/s41587-019-0036-z. PMID: 30778233

14. Almagro Armenteros J, Salvatore M, Emanuelsson O, Winther O, von Heijne G, Elofsson A, et al. Detecting sequence signals in targeting peptides using deep learning. Life Sci Alliance. 2019b;2: e201900429. https://doi.org/10.26508/lsa.201900429. PMID: 31570514

15. Savojardo C, Bruciaferri N, Tartari G, Martelli P, Casadio R. DeepMito: accurate prediction of protein sub-mitochondrial localization using convolutional neural networks. Bioinformatics. 2020;36: 56-64. https://doi.org/10.1093/bioinformatics/btz512. PMID: 31218353

17. Needleman S, Wunsch C. A general method applicable to the search for similarities in the amino acid sequence of two proteins. J Mol Biol. 1970;48: 443-453. https://doi.org/10.1016/0022-2836(70)90057-4. PMID: 5420325

16. Sim N, Kumar P, Hu J, Henikoff S, Schneider G, Ng P. SIFT web server: predicting effects of amino acid substitutions on proteins. Nucleic Acids Res. 2012;40: W452-457. https://doi.org/10.1093/nar/gks539. PMID: 22689647

18. Sievers F, Wilm A, Dineen D, Gibson T, Karplus K, Li W, et al. Fast, scalable generation of high-quality protein multiple sequence alignments using Clustal Omega. Mol Syst Biol. 2011;7: 539. https://doi.org/10.1038/msb.2011.75. PMID: 21988835

19. Tamura K, Peterson D, Peterson N, Stecher G, Nei M, Kumar S. MEGA5: molecular evolutionary genetics analysis using maximum likelihood, evolutionary distance, and maximum parsimony methods. Mol Biol Evol. 2011;28: 2731-2739. https://doi.org/10.1093/molbev/msr121. PMID: 21546353
