# Supplementary material for: Multi-site fungicides suppress banana Panama disease, caused by Fusarium oxysporum f. sp. cubense Tropical Race 4
Source: PLoS Pathog. 2022 Oct 20;18(10):e1010860. doi: 10.1371/journal.ppat.1010860 (PMC9584521; doi:10.1371/journal.ppat.1010860)
Supplement: S2 Table — (PDF) [file ppat.1010860.s010.pdf]

S2_Table Inhibition of plate growth of IPO323

**Fungicide**	**EC_{50}**[a,b]	**EC_{90}**[a,b]	**MIC**[a,c]
Azoxystrobin	0.0125	0.0331	~0.0300
Pyraclostrobin	0.0013	0.0025	0.0052
Trifloxystrobin	0.0020	0.00371	0.00465
Epoxiconazole	0.0072	0.0083	0.0104
Tebuconazole	0.0319	0.0388	0.0520
Triticonazole	0.1182	0.1495	0.1875
Fluxapyroxad	0.045	0.12	0.46
Bixafen	0.047	0.15	0.51
Boscalid	0.25	0.48	~1.20
Carbendazim	0.14	0.18	0.22
Thiophanate	3.88	5.87	12.15
Thiabendazole	0.41	0.52	0.76
Captan[d]	30.37	38.51	~40.00
Chlorothalonil	0.11	0.21	~0.30
Mancozeb[d]	0.99	2.05	~3.00
CTAB	0.64	0.70	~0.80
Dodine	0.76	1.04	~1.67
C_{18}DMS	2.52	3.87	4.97
LMW chitosan[d]	610.00	937.50	1188.00
Copper[d]	483.1	925.70	~1081.00
Garlic oil	16.69	24.86	31.40

All values are given as µg ml^{-1} (ppm); [a]Graphically determined from non-linear regression curves; [b]EC_{50} and EC_{90} represents concentration at which colony formation is inhibited by 50% and 90%; [c]MIC (=minimal inhibitory concentration) represents concentration at which no colony formation is detectable; [d]FocTR4 more sensitive than IPO323
