# Supplementary material for: Multi-site fungicides suppress banana Panama disease, caused by Fusarium oxysporum f. sp. cubense Tropical Race 4
Source: PLoS Pathog. 2022 Oct 20;18(10):e1010860. doi: 10.1371/journal.ppat.1010860 (PMC9584521; doi:10.1371/journal.ppat.1010860)
Supplement: S3 Table — (PDF) [file ppat.1010860.s011.pdf]

S3_Table Putative fungicide targets in FocTR4

Target	Name	BLAST[a]	UniProt ID
Azoles			
Lanosterol 14α-Demethylase	Foc_Erg11/1	1e-162	X0JWG9
	Foc_Erg11/2	1e-169	X0LR56
	Foc_Erg11/3	2e-148	X0JM02
SDHIs			
Succinate dehydrogenase complex	Foc_Sdh2	9.1e-129	X0JUP8
	Foc_Sdh3/1	2e-07	X0JTM7
	Foc_Sdh3/2	1e-09	X0K132
	Foc_Sdh4	9.3e-08	X0JC07
Strobilurins			
Cytochrome b	Foc_Cytb	1e-163	A0A1A7TD23

[a]Values are the error probability *P* for NCBI BLAST comparison with homologous proteins in *S. cerevisiae*
