# Supplementary material for: Multi-site fungicides suppress banana Panama disease, caused by Fusarium oxysporum f. sp. cubense Tropical Race 4
Source: PLoS Pathog. 2022 Oct 20;18(10):e1010860. doi: 10.1371/journal.ppat.1010860 (PMC9584521; doi:10.1371/journal.ppat.1010860)
Supplement: S9 Table — (PDF) [file ppat.1010860.s017.pdf]

S9_Table Sequences of cloning primers

Name	Sequence (5’ to 3’)
SK-Fox-5	ACCGAGCTCGAGCCTGGTGGT
SK-Fox-6	*GGTGAACAGCTCCTCGCCCTTGCTCACCAT*GTTTGCTGACGATAGTGTTTACAAG
SK-Fox-8	CTGGCTCCAGTTGAGTTGCAAAC
SK-Fox-11	*ATCACTCTCGGCATGGACGAGCTGTACAAG*ATGTCCTACGATCAGTACAATCAGA
SK-Fox-12	*CGGTATTCCAGTAATCGCATCCGTAACCGC*CTACTTGTTGTTGTTGGCCACAGA
SK-Fox-13	GCGGTTACGGATGCGATTACTGG
SK-Fox-16	*GCGGAAAGTTTGCAACTCAACTGGAGCCAG*CCCAACTGATATTGAAGGAGCATT
SK-Fox-17	*TAAACGCTCTTTTCTCTTAGGTTTACCCGC*CCCGATCTAGTAACATAGATGACA
SK-Sep-16	ATGGTGAGCAAGGGCGAGGAG
SK-Sep-17	CTTGTACAGCTCGTCCATGCCG

Italics indicate sequence complementary with another DNA fragment, which allows homologous recombination in *S. cerevisiae*.
