# Supplementary figures and images for: Multi-site fungicides suppress banana Panama disease, caused by Fusarium oxysporum f. sp. cubense Tropical Race 4
Source: PLoS Pathog. 2022 Oct 20;18(10):e1010860. doi: 10.1371/journal.ppat.1010860 (PMC9584521; doi:10.1371/journal.ppat.1010860)

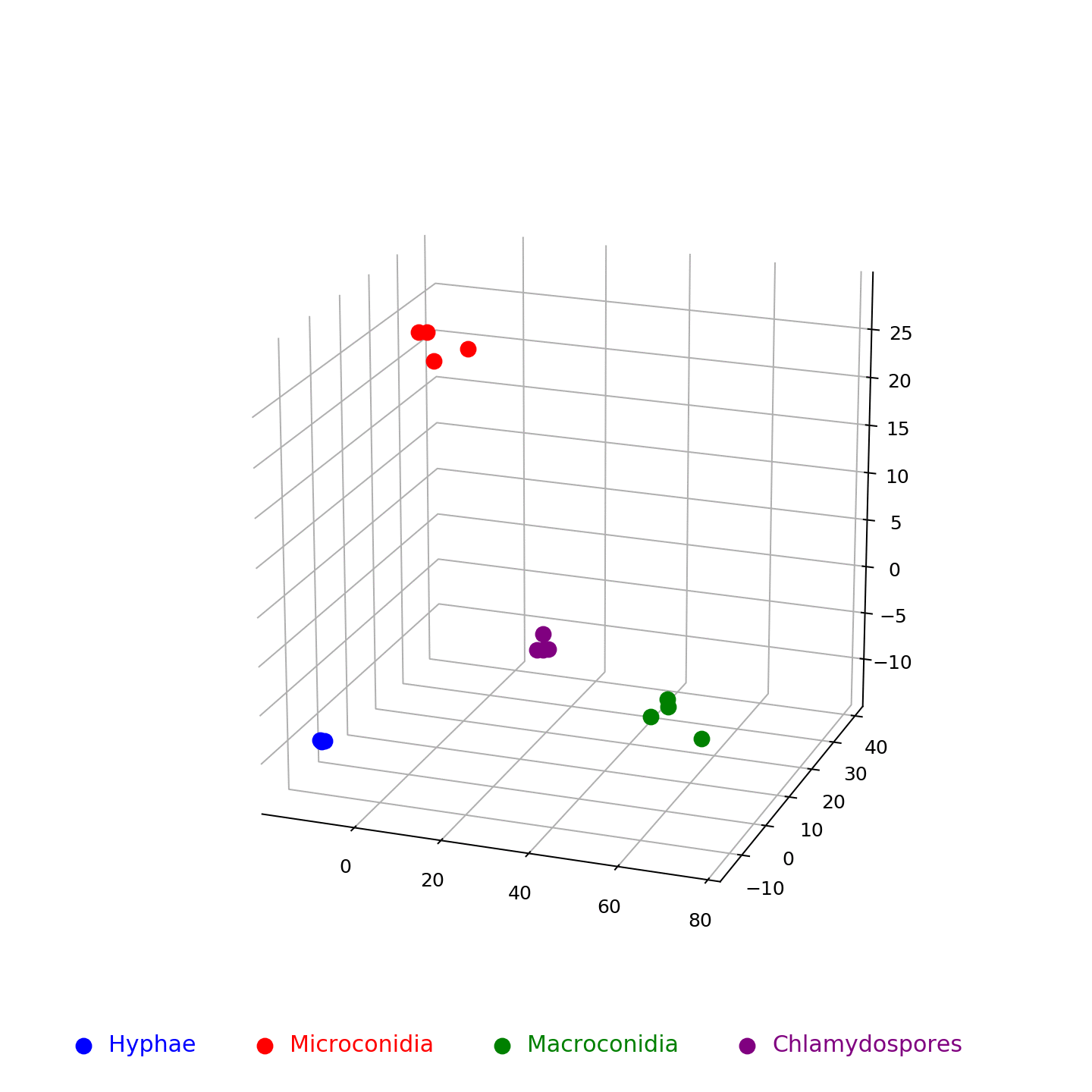

Supplement: S1 Video — (GIF) [file ppat.1010860.s019.gif]
