# Supplementary material for: Multi-site fungicides suppress banana Panama disease, caused by Fusarium oxysporum f. sp. cubense Tropical Race 4
Source: PLoS Pathog. 2022 Oct 20;18(10):e1010860. doi: 10.1371/journal.ppat.1010860 (PMC9584521; doi:10.1371/journal.ppat.1010860)
Supplement: Attachment — Submitted filename: Reply to Reviewers_Cannon et al_R.docx [file ppat.1010860.s020.docx]

**Point-by-point response to referee's comments**

***Reviewer #1****:*

*1. The concept and biogenesis of 'morphologically aberrant hyphae' / 'persister cells', as compared to other hyphae, needs to be experimentally substantiated.*

Response: We agree with the referee and in looking at the text and the data realise that we provided an ambiguous commentary. The way we phrased the previous text implied that persisters are always hyphae. However, we intended to say that cells, exposed to high concentration of azoles, SDHIs and strobilurins in media form morphologically aberrant hyphae. That hyphae are formed is not surprising, as the cells grown over days in this full medium, which initiates germination of the spores. However, that these hyphae survive such exposure makes the "persister" hyphae surprising.

In this revised manuscript, we show new images of such persisters in two Foc races, grown on plates (Fig. 2 g, h). In addition, we tested if spores, surviving high fungicide load for 10 days, switch into a particular morphotype (e.g. a spore form or hyphae). We did not find such morphological transitions (see new Fig. 6e) and therefore conclude that hyphae, as well as all spores can "persist" at high concentrations of fungicides. However, the detailed transcriptome analysis was done on persister hyphae, as this morphotype is most likely crucial for invasive growth during plant infection.

*2. Comparison with Zymoseptoria tritici should be removed*

Response: We disagree with the reviewer in this point. In comparing FocTR4 with *Z. tritici*, we make sure that the remarkable insensitivity to fungicides and the transcriptional responses of FocTR4 are not due to our experimental setup. This point is well illustrated when considering the transcriptional response of FocTR4 to fungicides. Our results show that FocTR4 responses to azole treatment with 660-times up regulation of the azoles-target gene Erg11. When going through the literature, we found up regulation of this gene in the presence of azoles in the range of 5-20-times. At this point we considered it possible that our sequencing techniques are more sensitive, or that other experimental differences account for this extreme transcriptional response in FocTR4. After we analysed *Z. tritici* in an identical way it became clear that this strong transcriptional response is unusual and is a hallmark of FocTR4.

We discussed this issue with the handling Editor and he agreed that the inclusion of *Z. tritici* strengthens the argument. However, we toned down the *Z. tritici* information and moved the Table of EC50 and other inhibitory concentrations from the main text to Supplementary Information. In doing so, we de-emphasis the data on *Z. tritici*.

*3. Testing another common F. oxysporum pathovar for a similar or different fungicide response would be highly desirable.*

Response: As this study is about Fusarium wilt in bananas, we suggested including another race of *Fusarium oxysporum* f.sp. *cubense*. We conferred with the handling Editor and he agreed that we test Race 1, known for its prior impact causing Panama disease on banana cultivar Gros Michel, in the fungicide sensitivity assays. Including Foc Race1 resulted in 21 new dose response curves (new Fig. 3; Table 1) and additional data, shown in Fig. 2. Including Foc Race 1 confirmed the insensitivity to major fungicides.

*Reviewer #2: Although several details need the author's attention, this work does not require additional experiments.*

*Reviewer #3: No major changes.*

***Part III – Minor Issues: Editorial and Data Presentation Modifications*** *Please use this section for editorial suggestions as well as relatively minor modifications of existing data that would enhance clarity.*

***Reviewer #1:***

*L 31/32: Should read ‘This could be due to insensitivity...’*
Response: We have corrected the text accordingly.

*L. 45: Should read ‘Zymoseptoria tritici...’*
Response: We have corrected the text accordingly.

*Fig. 1f: Explain on what basis the ‘principal component analysis of RNA-preparations’ was made.*

Response: We included a brief explanation and a new reference for the interested reader (new manuscript: ln 172 to 175).

*Fig. 2c: In the legend, it says ‘The central region show spores’, but doesn’t specifiy what kind of spores. The SEM picture shows hyphae and spore-like branches, however they neither look like microspores nor like macrospores.*

Response: In this revised version, we added an image for Foc Race 1 (Fig. 2f). In both images, the spores occasionally show septa, suggesting that they may resemble micro- or macroconidia. However, we do not feel comfortable to judge the "yeast-like" cells in the colony centre based on this morphological criterion alone. We added a note in the Figure legend to highlight the possibility that the cells are spores (Fig. 2b,c; 2e,f).

*Fig. 2f/ Fig. 6: Benzimidazoles seem to be effective against growing hyphae but not resting spores; therefore, they might be still be an option to be used in mixtures with multi-site fungicides.*

Response: This a good point, but testing fungicide mixtures is a future project. Indeed, we hope that our work provides the basis for such studies on fungicides mixtures. In this study, however, we focus on the effect of "pure" compounds and also search for fungicides that are able to "kill" spores.

*Fig. 3: Spores are resting structures which have a largely different gene expressing profile compared to growing hyphae, which makes it little surprising that expression profiles of stressed and unstressed hyphae are much more similar to each other.*

Response: As outlined in our response to the major point 1 by this referee, we realise that we have provided some confusing statements on persister hyphae in the first version of the paper. This revised manuscript is more focussed, and morphotype comparison with fungicide-treated hyphae has been removed.

*Fig. S5/S6: It is difficult to compare gene expression changes in response to fungicide treatments in two different fungi with different fungicide sensitivities. As expected, upregulation is observed in both organisms*

Response: Indeed, gene up-regulation changes is observed in FocTR4 and *Z. tritici*. However, the extent to which FocTR4 induces expression of particular genes is remarkable (e.g. 660-times increase of expression of Erg11; compare to 6.65-times in *Z. tritici*). By directly comparing a fungicide-sensitive fungus with FocTR4, it becomes obvious that some up-regulation is found in sensitive organisms; however, the FocTR4 response is, indeed, dramatic.

*L. 148ff: Based on a publication from Ploetz (2015a), that chlamydospores are most relevant for F. oxysporum infection, they performed microscopy of chlamydospore infections using GFP-GocSso1-expresssing cells. Later, they show that infection can also occur with similar efficiency when using micro- or macrospores or hyphae, although they all show a ‘distinctive transcriptional profile’. It would have been consequent to do microscopy also with these spore/ hyphal types to confirm the data with chlamydospores, otherwise I don’t see the point to show these experiments, and to do the RNAseq analysis.*

Response: Indeed, we agree with the referee that the infection strategy of FocTR4 and the role of all spore forms in this merit an extensive and detailed study. However, this study does focus on fungicide effects on Foc and the identification of potential cures. We needed to test the infectivity of spores to inform the spore mortality experiments (see Fig. 6).

We have opened the paper with *in planta* microscopy of chlamydospores and hyphae to "set the scene" and to engage the reader. The RNA seq experiments were done to support the morphological data with molecular results- both showing that we, indeed, prepared "pure" samples of all morphotypes. However, we realise that the functional annotation of the genes expressed in the morphotypes does not add to the study and, indeed, distracts the reader. We therefore removed former Supplementary Table 1 and Supplementary Fig. 1 (both showing functional annotation of expressed genes).

*L. 162: ‘To this end, we developed protocols to purify the 3 spore forms and hyphae (~94-100% pure; Fig. 1e)’ I can understand how the authors purified the 3 spore forms; but what about hyphae?*

Response: The method of purifying hyphae is fully explained in the Materials and Methods section, now included in the main text (ln 738-746)

*The hyphae shown in Fig. 1e are forming a mycelium that cannot be counted and applied in the same ‘numbers’ as the spores. Were the hyphae fragmented before counting and inoculation?*

Response: We admit that counting hyphae was a challenging task. Indeed, the hyphae were fragmented to allow accurate counting. The detailed method of hyphae purification is now included in the Materials and Methods (ln 738-746).

*l. 227: The authors asked and tested for the existence of possible mutations in fungicide target genes that would explain the resistance of persister cell. This seemed to be not necessary as the authors have shown that persister cells can return to normal growth of hyphae sensitive to fungicides.*

*Actually it is l. 267*

Response: We agree that the lack of inherited fungicide insensitivity confirms the absence of mutations in target genes. However, as written in the manuscript, we did the analysis of the target genes first. The experimental support was added after considering that mutations in non-target genes (e.g. transcription factors) could also provide genetically imprinted resistance. This is now more clearly written in the text

*l. 249: The principal component analysis of the RNAseq data should be explained in the results, at least I am not familiar with it.*

Response: We included a brief explanation and a new reference for the interested reader (new manuscript: ln 171 to 175).

*l. 582 (Discussion): ‘Moreover, we report that the ability to cope with high fungicide load is not genetically inherited‘. This sentence contradicts the conclusions in the Abstract (l..39): ‚innate resistance is genetically inherited in a sub-population of "persister" cells’.*

Response: We apologise for this crucial mistake The Abstract was corrected (now: Ln 39-41).

*- Fig. 6: The Live stainings of the different spore types in are interesting, but they are missing for ‚normal‘ hyphae after fungicide treatments. Furthermore, assays for germination rates would give a biologically more relevant and quantative result. One might have also considered to test the response of fungicide mixtures.*

Response: We appreciate that our study triggered the interest of the referee. We obtained solid quantitative data on spore survival and included these in the study. While interesting, testing mixtures of fungicides or quantitative germination assays of all 3 spore forms are major experimental tasks. We are in the "minor changes" section, so did not act on these points.

Regarding the third point (including hyphae in the LIVE/DEAD staining experiments), we tried to keep hyphae in water for 10 days. However, we found that even in the absence of fungicides the majority of hyphae die after 10 days (>60%). Thus, we could maintain hyphae only in medium. LIVE/DEAD staining experiments of hyphal growth in medium was already included in the manuscript (Supplementary Fig. S3a), so we did not perform more experiments. We explain this experimental limitation in the main text (ln: 451-454).

***Reviewer #2:***

*Line 44: The authors mention fungicide-detoxifying enzymes. This term is difficult and needs a clear definition or should be modified (see below)*

Response: We agree with this referee and have added a more detailed description in the results text and in the discussion.

*Line 47: The term pathogenicity is qualitative. In case quantitative differences of aggressiveness are to be compared, the correct term is virulence.*

Response: We thank the referee and have renamed the "pathogenicity assays" accordingly

*Line 108: The best way to control fungal pathogens …*

Response: We have corrected the text accordingly.

*Line 123-125: This statement does not make a lot of sense. If mutated genes are not expressed, there would be no phenotype. Moreover, not only mutations in target genes are relevant, but also mutations in transcription factors can cause overexpression of fungicide target genes. See the work by Kretschmer et al. (2009), PLoS Pathog. 5:e1000696. This work should be discussed and cited.*

Response: We are grateful for this comment and altered the text accordingly (ln 128-130).

*Line 138: Here MALCs are highlighted as effective chemistries against FocTR4. In line 48, captan is also recommended. Why is this compound not included here?*

Response: We are grateful for this comment and have altered the text accordingly (ln 143).

*Line 150: Replace infected by inoculated.*

Response: We have corrected the text accordingly.

*Lines 152-153: Delete sentence.*

Response: We do not think that the sentence can be deleted as we need it to justify why we addressed lipid droplets.

*Lines 157-158: Without hyphae, how can invasive growth occur? Delete sentence.*

Response: We agree and have corrected the text accordingly.

*Line 173: Spore suspension*

Response: We have corrected the text accordingly

*Line 174: …* *even distribution in the inoculated soil.*

Response: We have corrected the text accordingly

*Line 187: , and/or a hindering effect of soil: Explain or delete.*

Response: We have qualified this comment and included 3 supporting references. An more in-depth discussion is provided in the discussion.

*Line 204, and below: ED100 values are difficult to determine, due to asymptotic nature of graphs. Therefore ED90 values, in addition to ED50 values, are usually given and should be provided here as well.*

Response: We have determined EC90 values for all fungicides in FocTR4, Foc Race1 and IPO323; these values are now provided in Table 1 and Supplementary Table 2. The EC100 value was renamed MIC (=minimal inhibitory concentration), a term commonly used in studies in human pathogenic fungi.

*Lines 215, Table 1: Give two decimals only.*

Response: We wish to retain 3 decimals, as highly effective fungicides require this precision (e.g. Pyraclostrobin, EC50 in FocTR4 is 0.003- with 2 decimals it would be 0.00).

*Lines 236 onwards: To my understanding, certain hyphae activate mechanisms allowing them to grow in the presence of fungicides. They may be called persisters but could also be called fungicide-resistant/tolerant strains or resistant/tolerant hyphae. The alteration of morphology occurs only in the presence of fungicides, correct? And such alterations, as, for example, caused by azole fungicides, are known since long (Kang et al. 2001, Pest. Manage. Sci. 57:491-500), and it is not very surprising that azole applications affect plasma membrane function and, hence, cell wall rigidity. This applies also to the legend of Fig. 3A and should be clearly stated.*

Response: During this revision and reading the referee's comments (including this) we realise that we provided an ambiguous commentary. The way we phrased the previous text implied that persisters form hyphae. However, we intended to say that cells, exposed to high concentration of azoles, SDHIs and strobilurins in media form morphologically aberrant hyphae. That hyphae are formed is not surprising, as the cells grown over days in this full medium, which initiates germination of the spores. We have removed these comments now and clearly stated that hyphae are formed upon incubation in medium, supplemented with fungicides. These hyphae are aberrant, which, indeed, is un-surprising. However, that these hyphae survive exposure to such high fungicide concentrations is surprising, and we therefore prefer to call them persisters, as this reflects their survival capacity. In this revised version, we also include images of spores, incubated in fungicide-supplemented water for 10 days. The surviving cells did not undergo a morphological change, suggesting that the ability to cope with high fungicide loads is not restricted to hyphae. However, as these spores are expected to be dormant, the mechanism by which cells cope with fungicides is likely to be different from that found in persister hyphae of FocTR4.

We have attempted to describe our findings more fully and to better discuss the morphology and fungicide tolerance of spores and hyphae.

*Lines 251: In principally all cases I am aware of, fungicide resistance has only been tested in growing hyphae. In dormant structures (distinct spores), fungicide resistance is difficult to address. So, is it really surprising that hyphae, and not the spores tested, have fungicide resistance-related transcript profiles? The authors should discuss this.*

Response: Again, we apologise for a confusing description of our results in the first version of the manuscript. We did only analyse transcriptomes of hyphal persisters (grown and exposed to fungicides in medium), we did not address the transcriptional responses of spores in water. As referee 1 also struggled with this point, we decided to remove comparison of RNAseq results of fungicide-treated persister hyphae and the morphotypes from the manuscript.

*Lines 254 and following: Is the term inherited correctly used here? As an alternative, one could say that resistance is not mutation-based. The term inheritance suggests that sexual crosses have been made.*

Response: We disagree with the reviewer in this point, as per definition, "inherited" means "to receive from a parent or ancestor by genetic transmission" (Meriam-Webster Dictionary, https://www.merriam-webster.com/dictionary/inherit), which does not necessarily involve sexual crosses. However, we are now using "mutation-based" in the title of the Results chapter to make this point clearer.

*Line 271: … fluxapyroxad or 500 µg …*

Response: We have corrected the text accordingly

*Lines 323 onward: The authors discuss technical variations. Have RT-qPCR analyses been done to confirm the RNA-Seq data?*

Response: We did not do RT-qPCR, as transcriptome analysis is a much more reliable technique, which in our hands provided reproducible results.

*Line 340 onward: Here, the term detoxifying enzymes comes into focus. To my feeling, this should be defined more precisely. I fully agree that P450 enzymes belong in this group of detoxifying enzymes. But what about sterol biosynthesis enzymes? And what about transporters? These reduce intracellular drug concentrations, but is the term detoxification correct to describe this? Moreover, a methyltransferase with homology to a polyketide synthase (?) is thought to serve detoxification. The authors may wish to discriminate mechanisms conferring resistance more clearly.*

Response: We agree that the term "detoxifying enzymes" is very broad. We therefore defined the enzymes better using classifications published in the cited references Meyer, 1996 and Chen, 2020. We also removed the methyltransferase with homology to a polyketide synthase from Table 2 and all Supplementary tables. Sterol biosynthesis enzymes were included when they were annotated as cytochrome P450 monooxygenases (not in azole treated cells- here they are classified as pathway genes).

*Line 548: Replace the term cell suicide by the term cell death.*

Response: We have corrected the text accordingly

*Line 594: … this pump possibly participates …*

Response: We have corrected the text accordingly

*Line 621: … al. (2021) applied …*

Response: We have corrected the text accordingly

*Line 641: What does the term 'and the soil per se mean? Ion exchange complexity? Microbial complexity? Please define.*

Response: We defined this point according to the cited literature (ln 657-663) have corrected the text accordingly

*Lines 653-656: Not only captan, but principally all pesticides affect non-target organisms. This sentence should be deleted.*

Response: We do not wish to delete the sentence, but highlighted that other fungicides also affect non-target soil organisms (ln 672).

*Line 1251: … azoxystrobin or 500 µg …*

Response: We have corrected the text accordingly

*Line1329, Figure 5B: Green and red letters on a grey background are difficult to read.*

Response: The colouring of the writing reflects the colours given in the image. However, we overlaid the writing onto a black box, which improves the visibility (New Fig. 5).

***Reviewer #3:***

*Historically, Panama disease refers specifically to Race 1 and should be referred to instead as Fusarium Wilt of Banana*

Response: We appreciate this comment.

We consulted Wikipedia (admittingly a weak source), where it is clearly said " Panama disease (or Fusarium wilt) is a plant disease that infects banana plants" and "...Panama disease caused by the strain Tropical Race 4 (TR4)..." (<https://en.wikipedia.org/wiki/Panama_disease>).

Moreover, the CABI web page says "Fusarium tropical race 4 (TR4) is the common name of the soilborne fungus which causes Fusarium wilt in banana (Musa spp.), also referred to as Panama disease" (The invasive Species Compendium, <https://www.cabi.org/isc/datasheet/59074053>).

Finally, we searched peer-reviewed literature. We found confirmation of both statements in publications by Randy C. Ploetz (undoubtedly a leading expert of this disease). He says in 2000 (Plant Health Progress, doi: 10.1094/PHP-2000-1204-01-HM) "Panama disease, also known as Fusarium wilt of banana...". In Ploetz et al. 2015 (*Phytoparasitica* 43:283-293, DOI 10.1007/s12600-015-0470-5), he links Panama disease to TR4 in several places and most strikingly says in the legend of Figure 2: "...isolates recovered from Cavendish plants affected by Panama disease during the September 2014 survey were of tropical race 4 (TR4)...".

Thus, the entry in Wikipedia and the cited publications by Ploetz consistently state that (a) Panama disease and Fusarium Wilt of bananas are synonymous descriptors and (b) that Panama disease is caused by TR4. Therefore, we feel that we should keep our wording, as it is in agreement with published literature.

*Line 31 This could be due "to" insensitivity of the pathogen to fungicides and/or soil application per se.*
Response: We have corrected the text accordingly

*Line 44 Comparison of gene expression in FocTR4 and "the fungicide sensitive" - "Zymoseptora" tritici.....*

Response: We have corrected the text accordingly

*Line 48, At what concentration was captan and lipophilic cations effective?*

Response: We have added the effective concentrations of captan (20 µg ml^-1^) and the lipophilic cations (200 µg ml^-1^) to the abstract.

*Line 64, the use of "unusual hyphae" is vague.
Please consider adding in a better descriptive term for the hyphae.*

Response: As outlined above, we were not clear in our description of persisters. All expression studies were done in "persisting" hyphae, grown in fungicide-supplemented medium. The surviving spores did not turn into hyphae (new Figure 6e) and may have other mechanisms that allow them to cope with the fungicide load. We changed the text to account for this in the manuscript. *Line 142: What isolate of FocTR4 is being used and where was it acquired (please state in the methods section)*

Response: We used FocTR4 strain II5 (NRRL#54006, isolated from Musa spp. in Indonesia) and, in this revised version, added Foc Race1 strain CR1.1.A, VCG0120, isolated from Musa spp. in Costa Rica). This information is in Materials and Methods (now moved from Supplementary Information to the main text, ln 701-710 and all M& M up to ln 1118).  *Line 143 I'd suggest "fluorescent-tagged" version of.. for clarity*

Response: We have corrected the text accordingly

*Line 177-178 Any reference for image analysis?*

Response: We developed this method and described it in full in Materials and Methods

*Line 179 Replace “marker” with “indication”*

Response: We have corrected the text accordingly

*Line 190 We included the Fungicide- sus"c"eptible*

Response: We have corrected the text accordingly

*Table 1 Include a column indicating groupings of fungicides to 7 main classes*

Response: We have added the information to Table 1

*Fig 2 Change y-axis of all graphs to maximum 120*

Response: Figure 2 has changed and now includes data for Foc Race1. Extending the y-axes to 120% adds "empty information" and we therefore all y-axis graphs were cut at above 110%. The only exception is copper sulfate, where >130% is given, as low concentrations of copper appears to have a stimulatory effect on FocTR4 growth.

*Line 237 "with strong inhibition at low concentrations, but sparse growth at higher concentrations" paradoxical growth.*

Response: We have corrected the text accordingly.

*Line 237 Clarify why epoxiconazole not included in the statement but included in the test and succeeding discussion*

Response: We apologise for this and added epoxiconazole to the text

*Line 247 please add additional description for the morphologically aberrant hyphae, such as swollen, deformed, ect.*

Response: We have adjusted the text accordingly.

*Figure 3a Please add a control growth image for comparison.*

Response: In this revised version, Figure 3 has changed and 3a is now Supplementary Figure 3b. We now provide the requested control image. *Line 281 Remove “,” before (Steinhauer et al., 2019)*

Response: We have corrected the text accordingly.

*Line 302 Remove “.” after (L84W. Fig. 4b)*
Response: We have corrected the text accordingly.

*Line 305 Replace “.” with “and” between Foc_Erg11/3 and Foc_Sdh3/2.*

Response: We have corrected the text accordingly *Line 305 Indicate which gene is for azole and which is for SDHI resistance. Can just add “respectively”*

Response: We have corrected the text accordingly *Line 307 Where those untreated control cells hyphae? Please specify*

Response: We inoculated microspore solutions in PDB, either supplemented with the solvent methanol or with the fungicides. These growth conditions result in the formation of hyphae. We state this now in the text.

*Line 384 up-regulation of Foc_Sdh3/2 subunit strongly suggests SDHI-tolerance. Further molecular characterization is needed for a cause/effect relationship.*

Response: We have corrected the text accordingly  *Line 430: “**Tetramethylrhodamine, methyl ester (TMRM) dye signal” (or specify in the methods)*

Response: We have corrected the text accordingly

*Line 455 Change color of dot from red to black*

Response: We have corrected the text accordingly

*Line 471 Please include stat analysis used for Fig 6g*

Response: Fig. 6g is now part of Supplementary Fig. S6 and all statistical description is included in the figure legend (as it is for all other statistics in this paper)  *Line 495 Please elaborate on “obvious health differences from plants treated with water”*
Response: We have made a mistake here by omitting the word "no" (apologies). We corrected this mistake in the revised version.

*Table 2: Please include a column reporting differences in transcriptomic levels*

Response: This information was already present in Table 2, but the title of the column was displaced. We have corrected this now.

*For section: “All three FocTR4 447 spore forms survive treatment with most fungicides.” Were any germination tests run to confirm if any of the fungicides blocked germination?*

Response: We did not address germination, as we aimed to find fungicides that kill all spore forms, rather than preventing this developmental step.

*Line 502 Please clarify if day 0 and day 7 is after inoculation*

Response: We have corrected the text accordingly (ln 509) *Line 508 There is no Fig 7d*

Response: We have made a mistake here, it should be Fig. 7b. We have corrected the text accordingly. *Line 559: see above comment about ‘aberrant hyphae’.*

Response: We have added a brief descriptor of the persister hyphae to the text accordingly .

*Line 687: Fungal strains (FocTR”4”,... please list the source of the strain used*

Response: We used FocTR4 strain II5 (NRRL#54006, isolated from Musa spp. in Indonesia) and, in this revised version, added Foc Race1 strain CR1.1.A, VCG0120, isolated from Musa spp. in Costa Rica). This information is in Materials and Methods (now moved from Supplementary Information to the main text, ln 701-705).  *Line 738 Please include at what temperature was the phytotoxicity tests were performed considering tropical conditions for growing bananas.*

Response: We performed the phytotoxicity experiments (leaf assay, root drench assay) at 27 ± 1ºC, which reflects the optimum mean temperature for banana production world-wide (Varma & Bebber, 2019, Nature Climate Change, 9:752-757). We cited this reference in the revised manuscript. *Line 743 - 745: Please include more detail on how the fungicide was applied, what time frame.*

Response: We have moved the Supplementary Methods to the main text and extended the description of fungicide application to plants (ln 1073-1986).
