# Supplementary material for: Multi-site fungicides suppress banana Panama disease, caused by Fusarium oxysporum f. sp. cubense Tropical Race 4
Source: PLoS Pathog. 2022 Oct 20;18(10):e1010860. doi: 10.1371/journal.ppat.1010860 (PMC9584521; doi:10.1371/journal.ppat.1010860)
Supplement: Attachment — Submitted filename: Response to Referee and Technical team.docx [file ppat.1010860.s021.docx]

**Point-by-point response to referee's comments and Technical Check requests**

No response to reviewer #1 is required.

**Reviewer #2:** 
*Reviewer:* *The only general suggestion is to modify the text with respect to the many times the word “we” is used (six times in the paragraph lines 179-192; seven times on page 7; there are several examples). This I found disturbing as it takes away the focus from the data. The authors may wish to re-consider. The inclusion of ED90 values is appreciated.*
Our response: We appreciate this comment and carefully reviewed the text. We removed 30% of "we", which led to minor changes (see highlights in red).

*Reviewer:* *In the Introduction, the authors explain what chlamydospores and macro- and microconidia are and how they infect their host plant (Lines 96 – 105). I suggest deleting this section, as plant pathologists should know and not need this information.*

Our response: We appreciate the referee's comment. However, our introduction only mentions the existence of micro- and macroconidia (ln 94, ln 1370). As chlamydospores are thought to be of major importance in *Fusarium* infection and thus are in the focus of most of our plant-related experiments, we introduce chlamydospores more extensively. We feel that this is important and appropriate, as readers of PLOS Pathogens are not only plant pathologists, but those interested in animal, protozoan and other pathogens. We think that these readers would appreciate these few words of introduction. Thus, we do not wish to change the introduction.

*Reviewer:* *Lines 122-123: … related to the mode(s) of action of such fungicides within …*

Our response: We have amended the text (ln 123).

*Reviewer: Line 125: … enzyme catalyzing an essential process.*

Our response: We have amended the text (ln 125).

*Reviewer: Line 139: “We compared FocTR4 and Z. tritici transcriptional responses”: Why were FocR1 or any other fungicide-sensitive Foc strain not included?*

Our response: We have provided a rational for focussing our study on FocTR4, as this pathogen provides the current threat to Cavendish banana production (ln 139-141).

*Reviewer: Line 158: 7 days post inoculation.*

Our response: We have amended the text (ln 160).

*Reviewer: Line 183: We inoculated …*

Our response: We have amended the text (ln 185).

*Reviewer: Line 228 – Table 1: Would it make sense to add standard deviations? The authors give three decimals to indicate the preciseness of the data shown. However, SDs better reflects the preciseness.*

Our response: We appreciate this request. However, we determined the given concentrations from non-linear regression curves that were based on repeated experiments. Thus, while each data point in the graphs has a standard deviation (curves given in Figures), the linear regression curve does not provide these values. Moreover, adding more data to Table 1 would obscure the message. The reason why we added 3 decimal places is not precision, it is the only way to include the EC50 value for Pyraclostrobin, acting on FocTR4 (value: 0.005, see Table 1).

*Reviewer:* *Line 241: … most FocTR4 formed hyphae... Do the authors mean: … most FocTR4 microconidia formed hyphae?*

Our response: We have amended the text (ln 243).

*Reviewer:* Line 324: This suggests that strong …

Our response: We have amended the text (ln 326).

*Reviewer:* Line 559: FocR1

Our response: We have amended the text (ln 562).

*Reviewer:* Fig. 8b: What does plant health (%) mean? Does it mean %age of plants without disease symptoms? The authors call it a subtle phenotype (line 536), so the term plant health (%) may not be fully appropriate.

Our response: We added a short explanation of the term "plant health" to the Methods (ln 1072-1074) and in the Figure legend of Fig. 8b (ln 1793). We also removed the term "subtle" from the main text (ln 538).

**Technical Check Team:**

*Technical Check Request:* Please ensure that all author names are spelled correctly in the manuscript file, including the use of a middle name/initials, and any special characters (accents, umlauts, etc.). Please place first/given names before last/family names. All authors must have a first and last name. We noticed that " Sarah Jane Gurris" is spelled differently between the manuscript file and submission form. Please be aware that correct spelling and formatting of author names in the article file is vital for accurate indexing in PubMed.

Our response: We corrected the manuscript file accordingly.

*Technical Check Request:* We do not publish any copyright or trademark symbols that usually accompany proprietary names, eg (R), (C), or TM (e.g. next to drug or reagent names). Please remove all instances of trademark/copyright symbols throughout the text, including DEAD™ on line no 932.

Our response: We apologise for this mistake; the manuscript was corrected accordingly.

*Technical Check Request:* We have noticed that you have a list of Supporting Information legends "Table S9 - Sequences of cloning primers" in your manuscript. However, there are no corresponding files uploaded to the submission. Please upload them as separate files with the item type 'Supporting Information'.

Our response: We understand that all Supporting Tables (including Table S9, so far included in the main Supporting Information file) should be up-loaded separately. Thus, we have removed all Supporting Tables from the Supporting Information file and up-loaded them separately, using the item type "Supporting Information". We have updated the "Supporting Information contents list" in the Supporting Information file and in the Article file.

*Technical Check Request:* We have noticed that you have uploaded " Data S1_Cannon et al.xlsx" file as supporting information but you have not included them in the list of legends. Please add a citation of this SI data files into the list of legends of your supporting information files.

Our response: We have listed the Supporting Information file Data S1 in the list in the main text (ln 1490-1491) and in the Content list of the Supplementary Information file. We also included a note in the Data availability statement, saying " Numerical data and statistical analysis are provided in the Supplementary Information file Data S1. "

*Technical Check Request:* Please amend your detailed Financial Disclosure statement. This is published with the article, therefore should be completed in full sentences and contain the exact wording you wish to be published.

Our response: We have replaced the wording in the respective box during the submission process. We now say: " This work was supported by the Biotechnology and Biological Sciences Research Council (https://www.ukri.org/councils/bbsrc/; grants: BB/N020847/1, led by Dr. Dan Bebber, with SG and GS, and in association Prof. Gert Kema, Wageningen, The Netherlands; BB/P018335/1, led by Prof. Gero Steinberg, with SG). The funders had no role in study design, data collection and analysis, decision to publish, or preparation of the manuscript."

*Technical Check Request:* Some material included in your submission may be copyrighted. According to PLOS’s copyright policy, authors who use figures or other material (e.g., graphics, clipart, maps) from another author or copyright holder must demonstrate or obtain permission to publish this material under the Creative Commons Attribution 4.0 International (CC BY 4.0) License used by PLOS journals. Please closely review the details of PLOS’s copyright requirements here: PLOS Licenses and Copyright. If you need to request permissions from a copyright holder, you may use PLOS's Copyright Content Permission form.

Please respond directly to this email and provide any known details concerning your material's license terms and permissions required for reuse, even if you have not yet obtained copyright permissions or are unsure of your material's copyright compatibility. Once you have responded and addressed all other outstanding technical requirements, you may resubmit your manuscript within Editorial Manager.

Potential Copyright Issues:

[Data S1_Cannon et al]: Please confirm (a) that you are the photographer; or (b) provide written permission from the photographer to publish the photo(s) under our CC BY 4.0 license.

Our response: We confirm that all images included in this submission were acquired by the authors. No third-party image was included.

However, we also realise that your instructions for authors request only numerical data, underlying the statistical analysis provided in the paper, to be included in the Data S1 Excel file. We therefore now provide an up-dated Data S1 file (Excel), containing only the requested numerical data.
